# BALROG-ISO: a high-throughput pipeline for Bacterial AntimicrobiaL Resistance annOtation of Genomes-ISOlate whole genome

**DOI:** 10.17912/micropub.biology.001719

**Published:** 2025-11-05

**Authors:** Edward Bird, Victoria Pickens, Cassandra Olds, Kristopher Silver, Dana Nayduch

**Affiliations:** 1 Entomology, Kansas State University, Manhattan, Kansas, United States; 2 Arthropod-Borne Animal Diseases Research Unit, Agricultural Research Service, United States Department of Agriculture, Manhattan, KS, United States

## Abstract

BALROG-ISO is a Nextflow pipeline for automated analysis of whole genome sequences of bacterial isolates to perform taxonomic classification, genomic annotation, annotation of antimicrobial resistance genes (ARGs), and prediction of ARG origin (e.g., plasmid, chromosomal). A final summary report additionally offers a comprehensive and user-friendly visualization of key quality metrics and annotation results. BALROG-ISO minimizes command inputs and streamlines modular processes, enabling the concurrent analysis of more genomic samples while also reducing manual job submission and analysis inconsistencies. Overall, BALROG-ISO is an adaptable workflow pipeline optimized for a One Health approach to the exploration of antimicrobial resistance in bacterial genomes.

**Figure 1. Diagram of the BALROG-ISO workflow.**
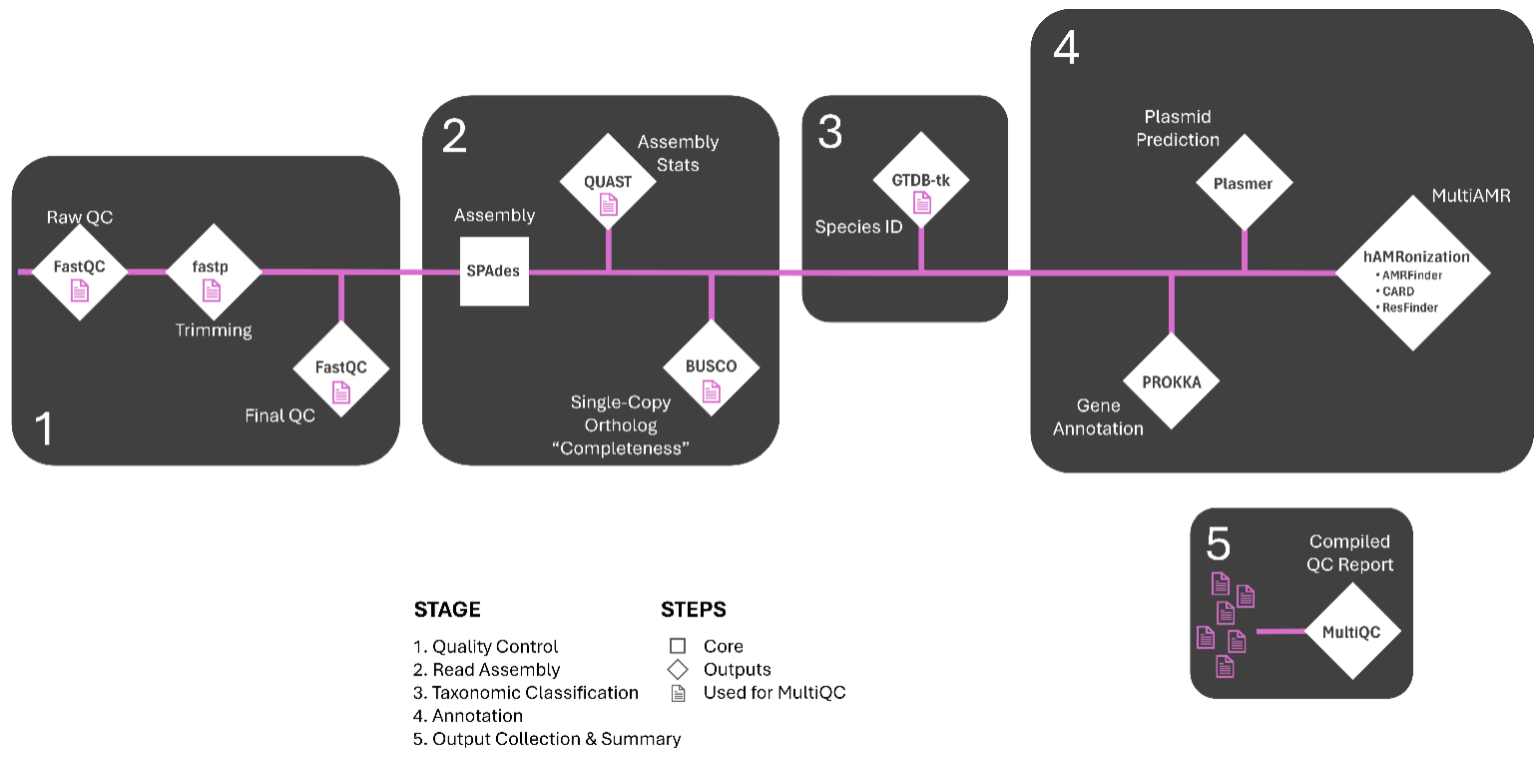
Outputs from Quality Control, Read Assembly, and Species Identification are summarized in a MultiQC report. Antimicrobial resistance (AMR) results are consolidated into a single report using hAMRonization. Prokka and Plasmer generate outputs on a per-sample basis. Stages 1, 2, 3 & 4 are run with a single command and the MultiQC report is generated by a secondary command on run completion.

## Description

BALROG-ISO (Bacterial AntimicrobiaL Resistance annotation of Genomes – ISOlate whole genome) ( https://github.com/edwardbirdlab/BALROG-ISO ) automates the assembly, species classification, and annotation of antimicrobial resistance genes (ARGs) from whole genome sequences of bacterial isolates. A rising and persistent threat to global health, antimicrobial resistance (AMR) decreases the efficacy of antibiotics for the treatment and prevention of bacterial infections. In accordance with the One Health approach, large-scale whole genome sequencing (WGS) of AMR bacterial isolates from clinical, food production, and environmental sources has become a primary tactic for the detection and monitoring of AMR emergence and prevalence of associated ARGs.

National and global initiatives release priority lists of pathogenic species to guide research and strategy development for combatting AMR (CDC 2019, WHO 2024). Current WGS analysis tools for bacterial isolates are therefore optimized for these priority species, resulting in a lack of efficient workflows for less commonly studied AMR species. However, WGS of “non-priority” species in the environment are essential for understanding their contributions to the establishment of AMR bacteria in clinical settings (Berendonk et al. 2015). Additionally, surveillance of AMR and associated ARGs in isolates has increased demand for high-throughput pipelines with streamlined workflows that significantly reduce the number of manual job submissions required from users while concurrently standardizing the data analysis process. To address this need, we designed BALROG-ISO, a reproducible Nextflow pipeline for surveillance of ARGs from WGS of isolated bacteria regardless of bacterial species or sample origin.

BALROG-ISO v1.0 (DOI: 10.5281/zenodo.15354071) consists of five major steps: (1) Quality Control, (2) Read Assembly, (3) Taxonomic Classification, (4) Annotation, and (5) Output Collection and Summary. BALROG-ISO is implemented using Nextflow, with each step isolated into modular processes that can be easily adapted or modified based on user requirements. The entire workflow runs with a single command, providing a streamlined and scalable approach. Dependency management is handled through Docker containers, ensuring consistent and reproducible results across various computing platforms. Initially, sequencing reads are trimmed to remove Illumina or Element adapters and low-quality bases. Cleaned reads are then assembled into contigs, which are subjected to taxonomic classification. Assembled genomes undergo both general structural and functional annotation, as well as antibiotic resistance gene (ARG) annotation. Finally, key quality metrics and annotation results are aggregated and visualized in an intuitive, user-friendly summary report.

During Quality Control, quality assessment of raw sequencing reads is performed using FastQC v0.12.1 ( https://www.bioinformatics.babraham.ac.uk/projects/fastqc/ ) to generate summary statistics and diagnostic plots. Human sequences are masked from raw sequencing data using the Human Read Removal Tool (HHRT) v2.2.1 ( https://github.com/ncbi/sra-human-scrubber ) with database version 20250325v2. Reads are subsequently processed with fastp v0.20.1 ( https://github.com/OpenGene/fastp ) for adapter trimming and removal of low-quality bases. By default, Illumina adapter sequences are removed; however, Element Biosciences adapters can be trimmed using the '--sequencing_adapter_type aviti' option. Custom adapter sequences may also be specified using '--sequencing_adapter_type custom' in combination with '--custom_sequencing_adapter_r1' and '--custom_sequencing_adapter_r2'. Users can modify trimming parameters, including the minimum average quality threshold ‘--fastp_q’ (default: 20) and the minimum read length ‘--fastp_minlen’ (default: 100). Following trimming, the cleaned reads are re-evaluated with FastQC v0.12.1 to verify improvements in quality and ensure suitability for downstream analyses.

Quality-controlled reads are assembled *de novo* using SPAdes v3.15.5 ( https://github.com/ablab/spades ) in ‘--isolate’ mode, which is optimized for isolated microbial genomes and provides improved assembly quality with reduced runtime. SPAdes automatically selects appropriate k-mer sizes based on the input read length, optimizing assembly parameters for each dataset. The resulting assemblies are evaluated using QUAST v5.2.0 ( https://github.com/ablab/quast ), which reports key assembly metrics including total length, number of contigs, N50, and GC content. To assess completeness, assemblies are analyzed with BUSCO v5.8.2 ( https://gitlab.com/ezlab/busco ) using the ‘bacteria_odb10’ lineage as the default reference set. Users may specify an alternative lineage by setting the ‘--busco_lineage’ parameter to specify the BUSCO set to use.

Assembled genomes are taxonomically classified using the Genome Taxonomy Database Toolkit v2.4.0 (GTDB-Tk; https://github.com/Ecogenomics/GTDBTk ) with the latest available GTDB reference release (Parks et al. 2022) automatically retrieved by the pipeline. Classification is carried out using the ‘classify_wf’ workflow, which integrates average nucleotide identity screening, phylogenetic marker gene detection, and multiple sequence alignment. Genomes are then positioned within the GTDB reference tree using a maximum-likelihood approach, enabling accurate assignment to bacterial taxa based on evolutionary relationships and genome similarity.

For genome annotations, Plasmer v0.1-20220816 ( https://github.com/nekokoe/Plasmer ) classifies sequences as either plasmid- or chromosome-derived and predicts the likely taxonomic origin of plasmid sequences. Functional genome annotation is performed using Prokka v1.14.6 ( https://github.com/tseemann/prokka ), providing gene predictions and general feature annotation. To identify antimicrobial resistance genes (ARGs), three tools are employed: Resistance Gene Identifier v6.0.3 (RGI; https://github.com/arpcard/rgi ) using the CARD protein homology models (Alcock et al., 2023), AMRFinderPlus v4.0.19 ( https://github.com/ncbi/amr ), and ResFinder v4.6.0 ( https://github.com/genomicepidemiology/resfinder ). AMRFinderPlus and ResFinder models are also used to find point mutations and genes specific to species. These models can be utilized by specifying the species for the run, using the two parameters ‘--amrfinder_lineage’ and ‘—resfinder_lineage’ respectively. The outputs from all three tools are integrated into a unified, standardized report using hAMRonization v1.1.8 ( https://github.com/pha4ge/hAMRonization ), facilitating downstream interpretation and comparison.

Plasmer results are presented in tabular format, while the outputs from the three ARG annotation tools are consolidated into a single comprehensive table. Results from FastQC (raw and trimmed data), fastp, GTDB-Tk, BUSCO, and QUAST are aggregated and visualized using MultiQC v1.28 ( https://github.com/MultiQC/MultiQC ). This tool compiles the outputs into a unified, easy-to-interpret report, offering a comprehensive overview of data quality, assembly statistics, taxonomic classification, and genome completeness across all samples.

BALROG-ISO is a publicly-available Nextflow pipeline designed for high-throughput, streamlined analysis of AMR and associated ARGs from bacterial whole genome sequencing data. This pipeline supports short read, whole genome sequences from a wide range of bacterial taxa, offering a standardized and reproducible workflow suitable for AMR/ARG characterization originating from any bacterial species, although users may alternatively opt to analyze a target bacterial species through simple parameter adjustments. BALROG-ISO’s modular design enables easy customization, as well as the integration of additional or specialized analyses. Tool outputs are consolidated into comprehensive visual reports for users’ ease to more effectively perform quality control and high-level screening of genomic data. Additionally, individual sample reports are comprehensive and allow for deep insights into individual samples of interest. By integrating ARG annotation, gene origin prediction, and taxonomic classification into a single, user-friendly workflow, BALROG-ISO will streamline AMR surveillance efforts, and its taxonomic freedom strengthens One Health strategies to monitor and mitigate the spread of AMR in pathogenic and environmental bacteria alike.
